# e-health literacy, health-seeking behaviors, and perceived ease of use of digital health systems in outpatients

**DOI:** 10.1515/med-2026-1523

**Published:** 2026-09-08

**Authors:** Cansu Özbaş, Merve Tokatlı Doğan, Berkay Ceylan, Hakan Tüzün, Seçil Özkan

**Affiliations:** Department of Public Health, Gazi University, Faculty of Medicine, Ankara, Türkiye; Istanbul Provincial Health Directorate, Istanbul, Türkiye

**Keywords:** eHEALS, online health information seeking, e-health systems, mHealth applications, e-health literacy

## Abstract

**Objectives:**

This study examines the associations between e-health literacy, multidimensional health-seeking behaviors, and the perceived ease of use of e-health applications among individuals aged 18–43 years in Turkey.

**Methods:**

A cross-sectional survey was conducted among 847 outpatients aged 18–43 years in Ankara, Turkey. Participants completed the eHealth Literacy Scale (eHEALS) and the Health-Seeking Behavior Scale (HSBS). Multivariate linear and logistic regression analyses were performed to determine predictors of health-seeking behavior and system usability.

**Results:**

Higher e-health literacy scores were significantly associated with both online and professional health-seeking behaviors (p<0.001), but not with traditional health-seeking behavior. Participants with higher e-health literacy were also more likely to perceive national e-health applications as easy to use (aOR=1.070, 95 % CI: 1.038–1.104). Gender and education level were significant sociodemographic predictors of professional health-seeking.

**Conclusions:**

E-health literacy may be associated with greater engagement with professional health services. The findings underscore the need for targeted e-health literacy interventions, even among digitally engaged younger populations, to promote equitable access to health services and reduce digital disparities.

## Introduction

The rapid evolution of internet technologies has fundamentally altered the landscape of healthcare delivery and information accessibility. Traditionally characterized by a reliance on healthcare providers, the patient role has shifted towards becoming a “proactive” consumer of health information [1]. Consequently, online platforms have emerged as primary sources of medical guidance for individuals. Factors such as the desire for privacy, cost-effectiveness, and the ability to access tailored information efficiently have accelerated this trend. Furthermore, utilizing online health resources can enhance health outcomes by fostering more active engagement between patients and physicians [2].

However, the widespread availability of online information does not automatically ensure effective health management. To benefit from these resources, individuals require health literacy, which is defined as the capacity to access, understand, appraise, and apply health information to make informed decisions [3]. In the context of electronic resources, e-health literacy is defined as the capability not only to seek, find, understand, and appraise health information from digital sources but also to apply this acquired knowledge to address health problems [4]. Using digital tools for information seeking alone may not be sufficient to improve health; without adequate literacy, individuals often struggle to understand the information they receive or identify misinformation [5].

Possessing these literacy skills is a prerequisite for effectively navigating the complex landscape of health-seeking. E-health literacy is intrinsically linked to health-seeking behavior, a concept defined as any activity undertaken by individuals who perceive themselves to have a health problem or to be ill for the purpose of finding an appropriate remedy [6]. Health-seeking behaviors can be evaluated in three distinct categories: traditional, professional, and online methods. Traditional methods involve individuals seeking support for their health problems from family members, friends, neighbors, and non-professional circles. Professional methods involve seeking solutions by consulting professionals providing health services. Online methods, on the other hand, involve individuals using the internet to conduct health-related research through health websites, forums, social media platforms, and other digital resources [7]. Therefore, understanding the relationship between e-health literacy and these specific sub-dimensions is essential for predicting health outcomes.

The practical application of these online seeking behaviors is often shaped by the digital infrastructure provided by national health systems. In the specific context of Turkey, the digitalization of the health sector has accelerated following the Health Transformation Program initiated in 2003. Within the framework of public policies aimed at increasing patient satisfaction and effective service delivery, critical systems such as the Personal Health Record System e-Nabız and the Central Physician Appointment System MHRS have been developed as cornerstones of the digital health economy [8]. However, the existence of these advanced digital infrastructures does not ensure equitable access for all. Research indicates that e-health literacy levels significantly influence information consumption patterns and search outcomes, extending the “digital divide” into the health domain. Consequently, individuals with lower e-health literacy are often limited in their ability to utilize available internet resources effectively. They are more likely to report lower perceived ease of use of e-health applications [9].

To analyze these dynamics, this study focuses on Generation Y and Z adults aged 18–43 years. This demographic is of particular interest because they represent the most active internet users and primary adopters of digital health technologies. Although often described as “digital natives,” high overall digital use does not necessarily translate into high e-health literacy, and younger adults may still face difficulties in evaluating complex medical information or navigating formal e-health systems. Recent evidence suggests that despite their familiarity with technology, young adults may still lack the specific skills required to evaluate complex medical information or navigate formal e-health systems efficiently [10]. Although the relationship between e-health literacy and general information seeking is well documented, few studies have examined how e-health literacy simultaneously shapes multidimensional health-seeking behavior and users’ perceived ease of use of nationally implemented e-health systems.

Therefore, this study aims to examine the relationship between e-health literacy and multidimensional health-seeking behaviors, as well as the perceived ease of use of e-health applications among individuals aged 18–43 years in Turkey.

## Methods

### Sampling

The study population consisted of individuals aged 18–43 years who applied to various tertiary health care outpatient units, excluding emergency services, at Gazi University Hospital in Ankara, Turkey, between June 12 and 14, 2023. The age range of 18–43 years was selected to target Generations Z and Y; for this study, they were defined as individuals aged 18–28 years and 29–43 years, respectively, in line with existing generational frameworks [11]. The study focused on Generations Y and Z because these cohorts are widely recognized as primary users of digital technologies and online health information sources. Restricting the sample to these generations was intended to ensure a more homogeneous level of digital exposure and to allow a focused examination of e-health literacy and online health information–seeking behavior [12]. Information obtained from the hospital statistical unit indicated that approximately 4,000 outpatient applications were made by individuals in this age range during the three days. Based on this population size (n=4,000), the minimum required sample size was calculated as 843 using Epi Info with an expected prevalence of 50 %, a margin of error of 3 %, a design effect of 1.0, and a 95 % confidence interval. The inclusion criteria were being aged 18–43 years, being literate in Turkish, having internet access, and providing consent to participate. Participants were recruited using a consecutive sampling approach, and 847 individuals were included in the study, exceeding the calculated minimum sample size.

### Implementation

This study used a cross-sectional design. Data were collected through face-to-face interviews between June 12 and 14, 2023. The research was conducted in accordance with the ethical principles of the Declaration of Helsinki. Ethical approval was granted by the Gazi University Ethics Committee (Date: 18.04.2023, Code: 2023-642). Informed consent was obtained from all individuals included in this study, or their legal guardians or wards. The study was prepared and reported in accordance with the Strengthening the Reporting of Observational Studies in Epidemiology (STROBE) statement [13].

### Data

The questionnaire form consisted of question groups addressing descriptive characteristics, e-health literacy, health-seeking behavior, and the use of e-health applications. The descriptive characteristics included age, generation classification (Generation Z [18–28 years], Generation Y [29–43 years]), gender (Female, Male), marital status (Single, Married), presence of children (Yes, No), educational level (Literate/Primary School, Middle School, High School, Associate Degree or Higher), income group (Income less than expenses, Income equal to expenses, Income higher than expenses), and presence of chronic disease (Yes, No). Self-perceived health status was assessed using a 5-point Likert scale ranging from ‘Very Poor’ to ‘Very Good’ (1=Very Poor, 2=Poor, 3=Moderate, 4=Good, 5=Very Good). E-health literacy was measured using the eHealth Literacy Scale (eHEALS), originally developed by Norman and Skinner [4] and adapted into Turkish by Gencer [14]. The scale uses a 5-point Likert scale, with higher scores indicating greater e-health literacy. Gencer reported the internal consistency coefficient (Cronbach’s alpha) of the Turkish adaptation of the eHealth Literacy Scale as 0.863 [14]. Health-seeking behavior was assessed using the Health-Seeking Behavior Scale (HSBS), developed by Kıraç and Öztürk [7], consisting of 12 items rated on a 5-point Likert scale and evaluating online, professional, and traditional health-seeking behaviors. For the original HSBS developed by Kıraç and Öztürk, the Cronbach’s alpha coefficients were found to be 0.755 for the total scale, 0.726 for the online search sub-dimension, 0.720 for the professional search sub-dimension, and 0.736 for the traditional search sub-dimension [7]. The final question group focused on the use of e-health applications. In this study, the term ‘e-health applications’ refers to government-based digital health platforms widely used in Turkey, specifically the Central Physician Appointment System (MHRS) and the Personal Health Record System (e-Nabız). Regarding the use of these applications, participants were asked: “Do you experience difficulty while using these applications?”

### Statistical analysis

Statistical analysis was performed using IBM SPSS Statistics 31.0 software. Descriptive statistics for categorical variables were presented as numbers and percentages, while continuous variables were presented as means and standard deviations. Prior to analysis, self-perceived health status was recoded into three categories (Poor, Moderate, Good), and the outcome variable for logistic regression was coded as 0 (Difficulty experienced) and 1 (No difficulty experienced). Normality was assessed using the Kolmogorov–Smirnov test and visual inspection of histograms and Q–Q plots.

Linear regression analyses were conducted to identify factors associated with HSBS scores, while logistic regression analyses were performed to determine predictors of the perceived ease of use (no difficulty experienced) of e-health applications. The relationship between the eHEALS and the HSBS was also evaluated using the Pearson correlation coefficient.

For the linear regression models, univariate analyses (Student’s t-test and One-Way ANOVA) were first conducted to identify candidate variables. Variables with a p-value <0.25 in these univariate analyses were included in the multivariate linear regression model to control for potential confounders. The eHEALS total score was included as the primary independent variable in both univariate and multivariate models.

For the logistic regression analysis, a similar two-stage approach was employed. First, univariate logistic regression analyses were performed to calculate Crude Odds Ratios (OR). Variables with p-values <0.25 in the univariate analyses were included in multivariate logistic regression models. In addition, variables with potential theoretical relevance were retained regardless of statistical significance. Multicollinearity was assessed prior to model construction. Adjusted Odds Ratios (ORs) with 95 % confidence intervals were reported. Separate models were constructed to examine the associations between e-health literacy and different dimensions of health information-seeking behavior. No automated variable selection procedure (e.g., backward elimination) was applied. In all final models, a p-value of <0.05 was considered statistically significant.

## Results

A total of 847 participants were included in the study, with a mean age of 30.80 ± 7.32 years. The majority of the participants were female (53.7 %) and belonged to Generation Y (57.5 %). The detailed distribution of sociodemographic characteristics and health status of the study sample is presented in Table 1.

**Table 1:** Distribution of sociodemographic characteristics and health status.

	n, %
Gender	
Female	455 (53.7)
Male	392 (46.3)
Generation	
Generation Z	360 (42.5)
Generation Y	487 (57.5)
Marital status	
Married	397 (46.9)
Single	450 (53.1)
Presence of children	
Yes	370 (43.7)
No	477 (56.3)
Educational level	
Literate/primary school	34 (4.0)
Middle school	46 (5.4)
High school	319 (37.7)
Associate degree or higher	448 (52.9)
Income groups	
Income is less than expenses	188 (22.2)
Income equal to expenses	316 (37.3)
Income is higher than expenses	343 (40.5)
Presence of chronic disease	
Yes	257 (30.3)
No	590 (69.7)
Self-perceived health	
Poor	50 (5.9)
Moderate	251 (29.6)
Good	546 (64.5)
Use of e-health applications	
Yes	791 (93.4)
No	56 (6.6)
Difficulty in using e-health applications	
Yes	166 (21.0)
No	625 (79.0)

Table 2 presents the correlations between eHEALS total score and HSBS sub-dimension scores. A statistically significant positive correlation was found between e-health literacy and online health-seeking behavior (r=0.358, p<0.001), professional health-seeking behavior (r=0.172, p<0.001), and the total health-seeking behavior score (r=0.288, p<0.001). However, no statistically significant relationship was observed between e-health literacy and traditional health-seeking behavior (r = −0.064, p=0.078).

**Table 2:** The relationship between eHEALS total score and HSBS sub-dimension scores.

	eHEALS total score	
	r	p-Value
Online health-seeking behavior sub-dimension score	**0.358**	**<0.001**
Professional health-seeking behavior sub-dimension score	**0.172**	**<0.001**
Traditional health-seeking behavior sub-dimension score	−0.064	0.078
Health-seeking behavior scale total score	**0.288**	**<0.001**

Bold values indicate statistically significant results (p<0.05).

Table 3 presents the results of the multivariate linear regression analysis on factors associated with health-seeking behaviors. In the first model, e-health literacy was found to be significantly and positively associated with Online and Professional Health-Seeking Behaviors and the Total Score, but not with Traditional Health-Seeking Behavior. In the second model, in which demographic variables were adjusted, e-health literacy remained independently associated with online and professional health information-seeking behaviors, as well as with the total score. Gender was significantly associated with all sub-dimensions of health information-seeking behavior, with male participants consistently scoring lower than females. Age was a significant predictor for both online behavior and the total score. Marital status was significantly associated only with online behavior, whereas education level was associated solely with professional behavior. Finally, the presence of children, chronic disease, and self-perceived health status were significant predictors of traditional health-seeking behavior.

**Table 3:** Multivariate linear regression analysis of factors associated with health-seeking behaviors scale score.

	Online Sub-scale score		Professional Sub-scale score		Traditional Sub-scale score		HSB Scale Total score	
	β^a^	p-Value	β^a^	p-Value	β^a^	p-Value	β^a^	p-Value
Model 1								
eHEALS total score	0.244	**<0.001**	0.052	**<0.001**	−0.026	0.078	0.271	**<0.001**
Model 2								
eHEALS total score	0.237	**<0.001**	0.047	**<0.001**	NS		0.284	**<0.001**
Age	0.066	**0.006**	NE		NS		0.073	**0.008**
Gender (ref: Female)	−0.735	**0.008**	−0.358	**0.006**	−0.425	**0.013**	−1.451	**<0.001**
Marital status (ref: Single)	−0.903	**0.008**	NE		NS		NS	
Presence of children (ref: No)	NS		NE		0.799	**<0.001**	NS	
Educational level (ref: Literate/primary school)	0.356	0.076	0.243	**0.010**	−0.211	0.090	NS	
Presence of chronic disease (ref: No)	NS		0.464	**0.003**	NE		NE	
Self-perceived health (ref: Poor)	NS		0.315	**0.010**	NE		NS	
Income group	NS		NE		NS		NS	

^a^β: Unstandardized regression coefficient. NE: Variable not entered into the multivariable model. NS: Variable entered into the multivariable model but not statistically significant. Bold values indicate statistically significant results (p<0.05).

Table 4 presents the factors associated with the ease of use of e-health applications based on multivariate logistic regression analysis. After adjusting for sociodemographic and health-related variables, the likelihood of finding e-health applications easy to use was higher among those with higher e-health literacy scores. In the multivariate model, the odds of ease of use increased by 1.07 times for each unit increase in the e-health literacy score (aOR=1.070; 95 % CI: 1.038–1.104). Younger age was associated with a greater likelihood of reporting ease of use (aOR=0.968). Participants reporting good self-perceived health were more likely to perceive e-health applications as easy to use compared with those reporting poor health.

**Table 4:** Predictors of ease of use (no difficulty experienced) of e-health applications: bivariate and multivariate logistic regression analysis.

	Ease of use of e-health applications^a^	
	Crude OR (95 % CI)	aOR^b^ (95 % CI)
eHEALS total score	1.076 (1.044–1.109)	1.070 (1.038–1.104)
Age	0.944 (0.921–0.967)	0.968 (0.942–0.995)
Gender		
Female	Ref	
Male	1.218 (0.862–1.721)	
Marital status		
Single	Ref	
Married	0.544 (0.385–0.769)	
Presence of children		
No	Ref	
Yes	0.526 (0.373–0.743)	
Education level		
Literate/primary school	Ref	
Middle school	0.381 (0.104–1.394)	
High school	0.889 (0.284–2.786)	
Associate degree or higher	1.398 (0.448–4.365)	
Income group		
Income is less than expenses	Ref	
Income equal to expenses	1.195 (0.764–1.870)	
Income is higher than expenses	1.408 (0.899–2.207)	
Presence of chronic disease		
No	Ref	
Yes	0.632 (0.441–0.906)	
Self-perceived health		
Poor	Ref	Ref
Moderate	2.748 (1.389–5.436)	2.028 (0.911–4.513)
Good	3.730 (1.953–7.124)	2.466 (1.143–5.321)

^a^Outcome coded as 1=no difficulty experienced, 0=difficulty experienced. ^b^Variables included in the model but not retained in the final model: Marital status, presence of children, education level, and presence of chronic disease.

## Discussion

This study aimed to evaluate the associations between e-health literacy and health-seeking behaviors, as well as the perceived ease of use of e-health applications among individuals aged 18–43 years. The findings revealed that e-health literacy was significantly associated with online and professional health-seeking behaviors, as well as the ease of use of national e-health systems.

In the multivariate analysis, e-health literacy was positively associated with online and professional health-seeking behaviors and the total score. This finding suggests that e-health literacy is associated not only with information retrieval but also with greater engagement with professional health services. Interestingly, no significant relationship was found between e-health literacy and traditional health-seeking behaviors. This lack of association suggests that seeking advice from non-professional social circles is likely driven more by cultural norms and social proximity than by digital competencies. The positive associations with other dimensions are consistent with the literature. In a recent study conducted with Chinese university students, it was reported that high e-health literacy increased access to hospital and physician services, as well as online information seeking [15]. Supporting this global pattern, a study of Brazilian undergraduate students found that the eHEALS scale is a predictive factor of digital health information-seeking behavior, with higher scores correlating with a greater likelihood of seeking health information [16]. Similarly, research involving Portuguese university students revealed that using the internet to research health information and consulting professional websites significantly increased the likelihood of having sufficient e-health literacy [17]. Furthermore, e-health literacy has been shown to encourage appropriate healthcare utilization and decision-making among Japanese adults [18]. Studies in Turkey further support this relationship; a significant positive correlation between e-health literacy and health-seeking behavior was identified [19]. Consequently, our findings indicate that e-health literacy may be associated with greater engagement with the professional health system.

Regarding demographic factors, gender was significantly associated with health-seeking behavior, with female participants scoring higher than males. This finding aligns with literature emphasizing women’s proactive role in health management. It has been noted that women utilize healthcare services more frequently due to their societal roles as “primary caretakers” and their engagement in regular maternal health checks. In contrast, men often delay seeking care [20]. However, it is essential to note that some studies reported no significant gender differences [21], 22]. The divergence in our results may be explained by the specific demographic characteristics of our sample (Generations Y and Z), a life stage during which women most intensively assume family health gatekeeping responsibilities. Thus, our results suggest that women in young and middle adulthood may utilize digital and professional health channels more actively due to their pivotal role in family health management.

When associations between sociodemographic factors and health-seeking behaviors were examined, distinct patterns emerged for education and parenthood. Our results indicated a significant association between higher education and professional health-seeking behavior. This suggests that higher education may be associated with a greater tendency to seek care through formal medical systems. This aligns with findings indicating that educated individuals are more likely to prioritize professional health care over other alternatives [23]. Conversely, the presence of children was identified as a significant predictor of traditional health-seeking behavior. This tendency can be attributed to parents’ inclination to utilize home-based care or consult their close social circle before visiting a hospital. Supporting this, a significant majority of caregivers administer home treatments or traditional remedies as a first response to their child’s illness before seeking professional medical advice [24]. Furthermore, the presence of a chronic disease was identified as a specific predictor of professional health-seeking behavior. Chronic conditions often require long-term management and strict adherence to medical regimens, necessitating frequent contact with healthcare providers. This finding is consistent with the literature, which indicates that individuals with chronic diseases are inclined to engage in active health-seeking behaviors to manage their conditions and improve their quality of life [21]. While chronic patients may utilize various information sources, our multivariate analysis highlighted that the professional dimension is the most significant pathway. This indicates that for individuals with established chronic conditions, reliance on medical expertise takes precedence over other information sources to ensure accurate disease management.

Furthermore, our study determined that higher e-health literacy scores were associated with higher perceived ease of use of e-health applications such as MHRS and e-Nabız. This finding is consistent with statements that high e-health literacy equips individuals with the competence to use complex digital health tools [25]. This relationship is also supported in the national context, where increased e-health literacy has been reported to positively influence perceptions of use of Turkey’s e-Nabız system [26]. Moreover, a strong effect of digital literacy on mHealth app usability (B=0.831) has been demonstrated, emphasizing the mediating role of “patient expertise” [27]. Additionally, a highly significant relationship (p<0.001) was found between e-health literacy and IT acceptance among women of reproductive age [28]. In our study, the finding that participants with high e-health literacy scores found the applications “easy to use” provides evidence that e-health literacy is a key factor in overcoming digital barriers. It is worth noting that although ease of use was assessed using a single-item measure, this approach reflects real-world user perception and has been commonly employed in health services research to reduce respondent burden.

Finally, self-perceived health status was identified as a strong predictor of perceived ease of use. Participants who rated their health as “Good” or “Moderate” were significantly more likely to find e-health applications easy to use compared to those with “Poor” health. This finding suggests that poor health imposes physical and cognitive burdens, complicating the use of technology. Although measured differently, this result aligns with the broader literature indicating that health problems hinder digital engagement. Specifically, it was demonstrated that medical conditions and functional limitations – which are often the underlying causes of poor self-rated health – act as significant barriers to internet and technology adoption [29].

Finally, although our sample consisted of Generations Y and Z – often labeled as “digital natives”- e-health literacy levels remained a decisive factor for system ease of use. This situation resonates with the “Digital Native Fallacy.” A systematic review conducted over a decade ago highlighted significant deficits in e-health literacy among college students [30], and current studies indicate that this landscape has not changed fundamentally. It has been emphasized that familiarity with technology is distinct from health literacy, with young adults still facing challenges in evaluating complex health data [10]. Therefore, our findings suggest that e-health literacy interventions should not be limited to older age groups but should also target younger generations, structured around “critical health literacy” principles.

### Limitations

This study has several limitations. First, the cross-sectional design prevents the establishment of causal relationships between variables; thus, results should be interpreted as associations. Second, data were collected from a single center, which may limit generalizability. Furthermore, participants were recruited from a tertiary care hospital over a three-day period using consecutive sampling. Therefore, the findings may not be generalizable to all young adults in Turkey, particularly those who do not seek care at tertiary healthcare institutions. Third, the data rely on self-reported measures, which may introduce recall bias and social desirability bias. In addition, e-health literacy was assessed using a self-reported instrument (eHEALS), which reflects perceived rather than objective skills; therefore, participants’ actual abilities may differ from their self-perceptions. Fourth, the perceived ease of use of e-health applications was assessed using a single-item measure, which may not fully capture the multidimensional nature of usability. Finally, the study was restricted to individuals with internet access, potentially excluding those with limited digital connectivity who may experience greater barriers to e-health use.

## Conclusions

This study demonstrates that higher e-health literacy was associated with online and professional health-seeking behaviors among young and middle-aged adults in Turkey. Individuals with higher e-health literacy were more likely to seek health information from digital and professional sources actively and to perceive nationally implemented e-health applications such as MHRS and e-Nabız as easy to use. These findings underscore that high digital exposure does not automatically translate into high e-health literacy; rather, specific competencies are required to engage effectively with digital health systems. Notably, traditional health-seeking behaviors were not significantly influenced by e-health literacy, highlighting the continued influence of sociocultural norms in shaping health-related decisions. Given the growing reliance on digital platforms in health systems, our results support the integration of targeted e-health literacy interventions, particularly among younger populations, to ensure equitable and efficient access to healthcare. Future studies employing longitudinal designs and multidimensional usability assessments are needed to explore causal pathways further and optimize digital health engagement.
